# Co-designing an interprofessional digital education resource on delirium: a student-led approach

**DOI:** 10.1186/s12909-024-06023-8

**Published:** 2024-10-10

**Authors:** Christine Brown Wilson, Tara Anderson, Margaret Graham, Jill Murphy, Gary Mitchell, Dympna Tuohy, Heather E. Barry, Pauline Boland, Matt Birch, Audrey Tierney, Patrick Stark, Arlene McCurtin, Laura Creighton, Elizabeth Henderson, Stephanie Craig, Hannah McConnell, Heather Guttridge, Lana Cook, Emma Cunningham, Geoffrey M. Curran, Alice Coffey

**Affiliations:** 1 School of Nursing and Midwifery, Queen’s University Belfast, Belfast, UK; 2 Department of Nursing and Midwifery, University of Limerick, Limerick, Ireland; 3 School of Pharmacy, Queen’s University Belfast, Belfast, UK; 4 School of Allied Health, University of Limerick, Limerick, Ireland; 5 School of Medicine, Dentistry and Biomedical Sciences, Queen’s University Belfast, Belfast, UK; 6 Center for Implementation Research, University of Arkansas for Medical Sciences, Little Rock, AR USA; 7 Center for Implementation Research, Health Research Institute, University of Limerick, Limerick, Ireland

**Keywords:** Co-design, Interprofessional education, Healthcare students, Delirium, Digital education

## Abstract

**Background:**

Interprofessional education is crucial for healthcare students to develop collaborative skills and provide effective patient care. However, opportunities for interprofessional learning are often limited in healthcare curricula. The present study aimed to engage students from different health professions in co-designing an educational resource on delirium recognition and management through an interprofessional lens and explore their experiences of this process.

**Methods:**

Two co-design workshops were conducted with students from medicine, nursing, pharmacy, and occupational therapy programmes at two universities across the island of Ireland. Focus groups were held following these workshops to explore students’ experiences of the co-design process. The workshops involved a range of activities, including theme generation, scenario development, resource creation (podcasts, simulations), and focus group discussions. Data from focus groups were analysed thematically.

**Results:**

A total of 19 students participated across the two workshops. Three themes were identified: (1) Relationship development, where students identified the benefits of co-creating the resource and valued the flexibility, collaboration, and social aspects of the co-design approach; (2) Interprofessional collaboration, which challenged students’ assumptions about other disciplines, fostered teamwork and communication, and highlighted the need for early and continuous interprofessional learning; (3) Professional growth, with students reporting increased confidence in managing delirium, working with other professions, and engaging in novel experiences like podcasting and simulation.

**Conclusions:**

The co-design process facilitated interprofessional collaboration, peer learning, and personal growth among healthcare students. Students appreciated the opportunity to co-create an educational resource while developing interprofessional skills. The study demonstrates the potential of co-design as a methodology for enhancing interprofessional education and promoting effective teamwork in healthcare.

**Supplementary Information:**

The online version contains supplementary material available at 10.1186/s12909-024-06023-8.

## Introduction

Delirium is characterised by an acute state of confusion which cannot be explained by a pre-existing neurocognitive disorder [1]. The prevalence varies depending on the patient population; while relatively uncommon in outpatient settings, delirium affects almost 20% of medical inpatients over the age of 75 years, and over 80% of mechanically ventilated patients [2]. Distress experienced in delirium has long-term psychological effects on patients and their carers [3]. Delirium is preventable [4], and despite being a growing global healthcare concern is frequently underdiagnosed [5], which may be attributed to a lack of awareness from healthcare professionals. For example, junior doctors across the UK and Ireland have demonstrated a lack of knowledge of the diagnosis and management of delirium [6, 7]. Nurses have also been found to lack knowledge of the diagnostic criteria for delirium, and have difficulty distinguishing between delirium, dementia, and depression [8]. The lack of awareness across disciplines highlights the need for improved delirium education. While there is a growing evidence base for the effectiveness of delirium education in practice [9], there is a lack of patient and public involvement [10] and a lack of research on how delirium education is delivered and understood by undergraduate healthcare profession students from disciplines such as nursing, medicine, pharmacy and occupational therapy [11, 12].

Recent National Institute for Health and Care Excellence (NICE) guidelines recommend multi-disciplinary delivery of treatment to effectively manage delirium [13]. Additionally, although evidence is limited, interprofessional education within delirium care has been found to improve patient outcomes in delirium [14]. Despite the benefits of interprofessional care, especially within the context of delirium, interprofessional student education remains limited, largely due to the challenges of scheduling, suitable venues and time involved [15]. However, digital educational interventions may present an opportunity to overcome some of these challenges by facilitating flexible learning to increase socialisation and collaboration in interprofessional student education [16].

The present study sought to evaluate healthcare profession students’ experiences of the co-design of a digital delirium education resource. The resource was co-designed as part of a wider study which aims to develop an interdisciplinary all-Ireland digital education resource to improve prevention, recognition and management of delirium among healthcare profession students [17]. The resource will provide healthcare profession students across the island of Ireland with information about how they can recognise, manage and prevent delirium with support from their interdisciplinary colleagues.

Co-design provides a catalyst for team-based processes in developing shared understandings and has been used effectively to engage students and academics in developing technology enhanced learning scenarios in higher education [18]. The co-design process also supports students in developing a greater understanding of design principles and enhances communication and collaboration with both educators and peers [19]. Specifically in healthcare, co-designed educational resources have been found to increase undergraduate nursing student knowledge of dementia and Parkinson’s Disease [20, 21]. Despite the highlighted benefits of co-design, evaluation of participants’ experiences of the co-design process is limited. Therefore, the present study aimed to explore student experiences by facilitating focus groups discussions at each stage of the co-design process.

## Method

### Research design

Co-design is based on the methodology of community based participatory research (CBPR) which aims to ensure research is an equitable process involving those who create knowledge through research and those who are intended as the knowledge users [22]. The present study used a co-design framework similar to other healthcare studies [23–25] adapted for a student population. In phase one, prior to the co-design workshops reported in this paper, the authors undertook focus groups with healthcare profession students [26] to provide triggers for facilitated team-based activities. The use of CBPR in this study was to ensure the development of an educational resource with shared accountability between the stakeholders [27]. A co-design framework was chosen for this study to ensure the student voice remained central to the project as the end users of the digital resource. Engaging students as partners in the research process was a priority and required honesty, inclusivity, trust reciprocity and shared responsibility [19, 28]. Therefore, the present study utilised relationship building activities within the co-design process taking place across two sites: the University of Limerick (UL), Ireland, and Queen’s University Belfast (QUB), Northern Ireland.

The aim of the workshops was to enable students to share their individual experiences of managing delirium, facilitate the co-design of educational content, and to collaboratively develop the interprofessional digital educational resource. Following the completion of the co-design process at each site, a focus group was held to explore students’ experiences of the co-design process.

### Recruitment

The study received full ethical approval from UL (Ref: 2023_06_20_EHS), affirmed by QUB (Ref: MHLS 23_122). Healthcare profession students were recruited to participate in the co-design process from UL (nursing and allied health (AHP)) and QUB (nursing, medicine, and pharmacy). Each university ran programmes in these areas and so had access to a range of students who may work together in healthcare environments to support people experiencing delirium. Students in both universities were emailed a study invitation through gatekeepers, following approval by the respective Heads of School. Details of the study and co-design workshops, approved/affirmed by the UL/MHLS ethics committees were sent to undergraduate students who had experience of caring for people with delirium- for example, medicine students had to have completed or be undertaking their care of older person module and nurses, pharmacists and AHP’s were expected to have completed at least one hospital placement. Students were asked to reply with an expression of interest which asked for details of experience of caring for a person from placement or family member experiencing delirium. Students were selected based on their experience of caring for people with delirium. The final selection ensured that the professions of nursing, medicine, pharmacy and allied health were represented. Participants were not selected according to gender or ethnicity.

### Co-design process

**Fig. 1:**
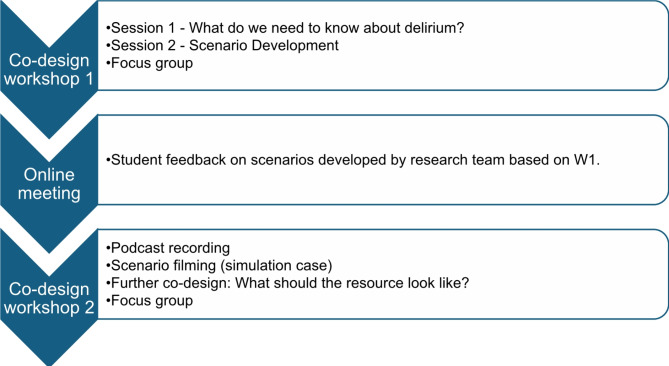
Co-design process. (Adapted from Santin et al., 2019 [23])

Co-design workshop one (W1) was held across a weekend at UL in September 2023. A total of 16 healthcare profession students from QUB (*n* = 12) and UL (*n* = 4) attended this workshop, and students were separated into small interdisciplinary groups (e.g. two nursing students, one medical student and one pharmacy student). Triggers from the focus group study data were used as prompts to guide activities [26]. During this workshop, students discussed their previous experiences with delirium, what is needed within delirium education, and developed potential clinical scenarios they may encounter. A catch-up meeting was held online via Microsoft Teams four weeks following W1. Students were presented with drafts of the scenarios, further developed by the research team based on W1, to be filmed in workshop two (W2) and asked to provide feedback. Three weeks following this catch-up meeting, co-design W2 was held over two days at QUB in November 2023 with 18 healthcare profession students from QUB (*n* = 12) and UL (*n* = 6). Figure 1 provides an overview of the co-design process. During W2, students participated in the creation of video and audio materials for the resource as well as further co-design activities regarding what the resource should look like and how to promote student engagement with the resource.

### Relationship building

Relationship building activities between the research team and students were incorporated throughout the course of the two co-design workshops. These included team lunches, dinners, and cultural activities (Irish music showcase, sightseeing, attending a sporting event). Relationship building facilitated more open and honest discussion and ensured students felt comfortable to express their views and experiences with both their peers and the research team. Relationship building was of particular importance given that students were recruited from five different Schools across two universities and therefore were likely to be unfamiliar with each other.

### Data collection

#### Artefacts

Data were collected from the co-design workshops in the form of artefacts such as Post-it^®^ notes, ranking exercises, whiteboards and flip chart paper; images of these may be viewed in the supplementary material.

*Co-design workshop 1* focused on theme generation. Within their groups, students first engaged in a ‘Stream of Consciousness’ task which involved creation of a word cloud based on the prompt ‘What is Delirium?’. Stream of consciousness is a timed writing activity where all ideas are welcomed without judgment [29] to support all members of the group to be involved. Next, students completed a prioritisation activity where each group analysed their answers and categorised their words under thematic headings (Supplementary Material S1). Each group presented their themes to facilitate discussion and develop consensus across groups on the key themes to be covered in the resource. Students also provided feedback on the specific delirium teaching they each experienced within their programmes. Session two began with a presentation of trigger material from the systematic review findings from Phase 1 of the wider study [30]. Students explored their own experiences of supporting a person with delirium and analysed this experience alongside the evidence from the systematic review. Within small interprofessional groups, students used this information to develop scenarios to enable healthcare profession students to understand how to recognise, manage, and prevent delirium in an engaging way (Supplementary Material S2).

*Co-design workshop 2* focused on resource development. An audio podcast was recorded on day one by a group of students (two nursing, one medicine, one pharmacy). On day two, two scenarios were filmed in a simulation lab while an ongoing co-design session was held throughout the day which students could drop in and out of depending on other activities. The second co-design session developed the features of the resource including a series of quizzes and encouraged students to engage with and review the learning embedded in the resource. As different groups of students joined the co-design session across the course of the day after filming/ podcasting, this workshop used an iterative process with students building on each other’s work. For example, one student identified questions for the resource with a later student identifying how a scenario could be linked to the questions, followed by a third student later in the session who developed the scenario with additional branching activities. Artefacts from the second co-design workshop may be viewed in the supplementary material (Supplementary Material S3). The final resource comprises an interactive website that starts with an overview of delirium and allows students to explore the assessment, management and prevention of delirium. Each section of the website houses interactive features such as drag and drop questions and answers, an interactive brain, podcasts by healthcare professionals and students and an end of unit quiz. These were all features identified as important for students to promote engagement in the co-design workshops. The final section on multidisciplinary care features the student led video of a case study co-designed in the workshops.

#### Focus groups

Focus groups were held at the end of each workshop to explore the student experience of the co-design process. Consent forms were signed prior to W1 with a reminder provided to students at the start of each focus group that their participation was voluntary, and they could elect not to be involved and withdraw at any time. The focus groups were audio recorded with the students’ permission and consent prior to each workshop and fully transcribed.

### Data analysis

Data were collected via a total of three focus groups facilitated by two members of the research team (CBW and TA). Each focus group lasted between eight and 40 min. At the beginning of each focus group, the purpose of the study and ground rules were established including the importance of adhering to ethical principles of confidentiality. The eight-minute focus group was to ensure the views of students engaged in the filming of the interprofessional scenario, during the main focus group discussion, were captured and was undertaken during their filming schedule. We also returned to these students following their filming schedule to ask if there was anything further they wished to add but no new insights were shared.

The focus group guide (Table 1) utilised open-ended questions to generate a discussion in addressing the research question. One of the researchers led the focus groups (CBW) with a second researcher observing and taking field notes (TA). The recordings were transcribed verbatim and thematically analysed [31]. This consists of data familiarisation, initial code generation, theme search, theme review, defining and naming themes, and report production. Transcriptions of each focus group were read and coded by one researcher (TA), with a second member of the team checking for meaning (CBW). The same researchers grouped the codes and refined the themes. Preliminary codes with examples of anonymised data were shared with members of the wider authorial team to sense check the analytical process by the first author. The final themes were discussed with a third member of the team (GM) to ensure congruence with the data.

A key element of qualitative research is reflexivity. As a large team it was important that all voices were heard, and consensus reached in decision making. The first author took leadership (CBW) in this process at regular meetings held before and after each codesign workshop with the whole team. Smaller groups were assigned responsibility for specific elements of the facilitation, organisation and delivery of each codesign workshop. The researchers engaged in the collection of focus group data (CBW/TA) met before and after each workshop to prepare and debrief.

**Table 1:** Interview guide

Interview guide
Welcome and Introductions
What has your experience of the co-design workshop been like?
What worked well about the co-design process?
Tell us what did not work so well?
What suggestions would you have in designing future co-design workshops?
How important has the social aspect been?
How did the structure of the group work?
What would you like to see happen next?
Is there anything else that you would like to add that we have not discussed?

## Findings

### Demographic details

Prior to the first co-design workshop, four UL students and twelve QUB students were recruited representing the professions of nursing, medicine, pharmacy, and occupational therapy (see Table 2 for demographics). All students attended W1. Between W1 and W2, a further four UL nursing students were recruited while two of the original UL students could not attend W2. Therefore, the second workshop was attended by the twelve QUB students and six UL nursing students. In total, 19 students engaged in focus groups across the project.

**Table 2:** Participant demographic details

Gender	Male	5
	Female	14
Health Profession Discipline	Medicine	4
	Pharmacy	4
	Nursing	10
	Occupational Therapy	1
Programme Level	Undergraduate	18
	Postgraduate	1
Year of Study	First	1
	Second	8
	Third	4
	Fourth	4
	Fifth	2

### Themes

Three themes were identified: Relationship development where students identified the benefits of co-creating the resource and what elements supported their relationships to develop; Interprofessional collaboration that focused on working together across disciplines and the impact such collaboration had on students; and Professional growth where students reflected on how the co-design process had supported them in developing confidence in caring for people experiencing delirium and working with other professions. These themes and their corresponding codes are presented in Table 3.

Participants have been coded in terms of the focus group (FG1/2/3), their university (U1/U2), programme of study (nursing/medicine/pharmacy/occupational therapy), and year of programme to facilitate identification of the range of perspectives evident across universities and disciplines. The students involved have read and approved this manuscript and have consented for their data to be presented in this way.

**Table 3:** Codes and categories generation

Relationship development	Interprofessional collaboration	Professional growth
Flexibility to co-create	Value of interprofessional collaboration	Learning together
Facilitated collaboration	Previous interprofessional experience	Confidence in managing Delirium
Efficient	Previous assumptions	Personal growth
Social aspect	Same goal	
Executive decisions	Lessons for future interprofessional learning	
Areas for improvement		
Hopes for future co-design		
Final product		

#### Theme 1 –Relationship development

Students explained how the process of co-design supported the building of relationships within the groups. The flexibility to co-create ideas, the collaborative approach within the workshops and the opportunity to socialise outside of the workshops were key elements that contributed to relationship development. Students also identified there was a natural conclusion to their involvement and a time for the research team to make decisions to move the process forward.

Students found the co-design process beneficial, in particular they valued the time and flexibility to co-create ideas:

“I like the fact it… wasn’t really rigid… we had like a freeness to come up with, and create, and actually think for ourselves’, ‘the structure… is very good and all the… organisers are very flexible in terms of time schedule” (F1U1Y3Nurs1).

Using a range of activities facilitated students in formulating ideas in a relatively short period of time:

“I think the speed at which we’ve got pretty concrete ideas already is impressive” (F1U2Y5Med1),

“From a time management point, I thought it was very well done that, like ideas were discussed properly and hashed out and not like rushed or anything” (F1U2Y2Pharm).

Students also discussed how the co-design process facilitated collaboration promoting interprofessional learning:

*“*I loved how if I had one idea, someone else, that would trigger something in their head which would then trigger something else in my head and it was like a constant stream of like ideas” (F1U2Y4Pharm),

*“*You always try to make sure that there was always one nursing student, one medic, and one pharmacy student in a group and that’s like it doesn’t give people an opportunity to sort of group together in their one discipline and then… think my ideas are correct because most of these people around me thinking the same thing… you were forced in effect to sort of exchange ideas and listen” (F2U2Y5Med).

Students identified the value of the relationship building activities across the co-design process:

“I think another thing that helped our relationships blossom out of this [the co-design process] is that fact that … we’ve also… done different things outside of this [the co-design process] to chat like to talk and I think that really makes it work well” (F2U2Y2Nurs),

“We all kind of got to socialise a bit before and like know each other a wee bit, it makes that when you go in to actually do stuff you’re all kind of ready to do it, and like the ice has already been broken which is kind of important” (F1U2Y4Med).

Following the first co-design workshop, students discussed suggestions for going forward which included the point at which decision making from the research team would be necessary as students felt it would be difficult to collaborate between the two workshops due to conflicting schedules:

*"Considering all the ideas that have been put forward today*,* I think*,* maybe*,* if there was like executive decisions made*,* as in okay we’re going to do a podcast…" "Maybe a little bit of direction now"* (F1U2Y3Nurs).

*“*It will probably be difficult… in the next couple of weeks to get anything as near productive as today was” (F1U2Y5Med2).

The importance of executive decisions was also discussed in terms of accuracy:

*“*If [the resource] is going to be rolled out in a widespread fashion there’s a lot of pressure for that to adhere to the guidelines and everything has to be so carefully done… you have to be so careful about making sure everything is accurate and all up to best practice” (F1U2Y5Med1).

Additionally, following the second co-design workshop students acknowledged that although these executive decisions were necessary, they still felt the process was student-led:

*“*I know that the script writing was done by yourselves, and I know [the researchers] thought about maybe us doing it but I think that could have maybe felt a bit disjointed. So, it’s about acknowledging that it’s co-working, it’s co-designed but there are maybe times when it’s best… for yourselves [the researchers] to take the lead” (F2U2Y5Med1).

Students were also able to see their ideas reflected in the final product, which underpinned how they had led the development of the resource:

“I just feel like that was translated over really well… the points we got together in our groups on weekend one… you took on board and meshed and blended really well to hit all those points in the scenario and do it in a nice, succinct, understandable way” (F2U1Y3Nurs1),

*“*it’s nice to see how similar the scenarios are to what we envisioned… and it’s not just been like over written… by seniors” (F2U2Y5Med),

*“*It’s been really nice to see the whole sort of process evolve and like to get to this stage [end of co-design workshop 2] now where we basically have a podcast, we have the videos which was just kind of seemed so far away whenever we were in Limerick [during co-design workshop 1]” (F2U2Y4Pharm),

Students made suggestions for improvement regarding the co-design process which included utilising the time together as a group more and involving more disciplines.

*“*Be interesting to add in a physio as well to see what they would have to say’ (F1QUBY2Nurs), "Even a dietitian as well” (F1U1Y2OT).

Students recognised the benefit of being central to the co-design process and expressed how the co-design process could improve future practice:

*“*Hopefully like in the future other topics… might start getting more input from students to change the whole system for the better… realistically we’re going to be the ones in the system for the next few generations like our opinion will matter a lot in the long run… it’s not just delirium that needs this [co-design], it’s loads of things*”* (F1U1Y3Nurs2),

Finally, students discussed how the relationships they had developed supported them in feeling comfortable working together on the end products of the co-design process. Students in W2 were divided into three streams of activity to ensure all students were involved: recording a podcast, engaging in the simulated scenario, and filming the case study. Students across all streams of activity worked together for the end result. Students engaged in the podcast discussed their experience, which was both intimidating and rewarding:

*“*It was good that the podcast went towards the end only because I think… for something like that you have to have an already established sort of relationship with the people that you do it with” (F2U2Y5Med2).

Despite feeling nervous, the students involved in the podcast recording reported feeling happy with the outcome and accurate timing of the recording within the workshop:

*“*I found the podcast really nice, and it was really good” (F2U2Y2Nurs),

*“*It did work in the end because people relaxed” (F2U2Y5Med2),

*“*It was nice to really hash everything that we’d already talked about out” (F2U2Y2Nurs).

The simulation stream, a small interprofessional group who were working with simulated patients to film a scenario of interprofessional working, discussed similar points:

*“*It was obviously nerve-wracking at the start because you don’t know anybody… but I just felt very comfortable… throughout the simulation*”* (F3U1Y2Nurs).

*“*It was good to have a real actor as well because it really, it feels realistic and even if you have like a dummy, there’s only so much you can say to the dummy… especially for delirium and the type of project we’re doing when it’s very unpredictable, I think that [the actor] really has enhanced the experience” (F3U2Y2Pharm).

Overall, the students engaged in the co-design process felt overwhelmingly positive about their involvement and were able to reflect on how building relationships was a key part of the process that enabled them to work effectively together to co-create the resource.

#### Theme 2 - interprofessional collaboration

Students discussed their experience of interprofessional collaboration during the co-design process as helping them to dispel previous assumptions of other disciplines and consider the ongoing value of interprofessional learning. Areas such as understanding other disciplines’ roles, improved communication and teamwork were identified as promoting collaboration leading to improved patient care. The impact of interprofessional learning within the co-design process also prompted students to reflect on how interprofessional learning might be better embedded into their curricula.

Students expressed surprise at the difference between their assumptions of other disciplines’ roles and their experience of collaborating with these disciplines as part of the co-design process. For example, students may only see part of the role they or other disciplines play:

“You’re going in with kind of an assumption that pharmacists just kind of… get the Kardex, they dispense and stuff like that and you don’t really know how much of a patient involvement they have” (F2U1Y3Nurs1),

*“*Helped me kind of see what nursing students would have done before like [medical students] only come in halfway through the situation and the nurses have already done all this background… and they’ve a better understanding of patients… it gave me a greater appreciation of like their knowledge basis*”* (F3U2Y4Med).

*“*You [doctors] just kind of like fly in… see a set of symptoms, you give a diagnosis and you’re kind of out the gap again whereas I think from talking with each of [the other students] on weekend one like you really got to see how patient focused you all actually are… so, it kind of tears down that unfortunate assumption” (F2U1Y3Nurs1).

In addition to challenging previous assumptions, students also felt the experience helped them to realise that all disciplines are working to achieve the same goal of patient care:

*“*It’s nice to see it is similar, that we do have the same goal” (F1U1Y2OT),

*“*we all have the same goal of the patient but like completely different areas… different kind of views and different experiences” (F2U1Y3Nurs2),

Interprofessional collaboration requires teamwork and communication, and all students identified these elements as integral to providing effective patient care:

*“*It makes it quicker to achieve the recovery state for the patient if there’s better communication” (F3U1Y2Nurs).

“Knowing what each role is… that helps make communication and teamwork a lot easier” (F3U2Y4Med),

“You need to have some kind of a working relationship, even just like knowing each other, because then things will get done better, like you’ll be able to actually properly communicate… everyone needs to be able to be used to communicating with the different teams” (F2U1Y3Nurs2).

Despite the benefits discussed, students acknowledged a significant lack of previous interprofessional learning opportunities across medicine, pharmacy, and nursing:

“We’ve never touched base really with pharmacists… unless you’re on the ward where you’re thrust into it” (F2U1Y3Nurs1),

“I just think we should have more inter[professional] links… cos we need to know what the other professions do and who we need to call and who needs to be there… for it to work a bit more streamlined” (F2U2Y2Nurs).

Medical students also discussed limited interprofessional learning:

“We always used to do it with pharmacy students in first year… just turned into a case of we were sat in a group trying to solve like basic chemistry questions… there was no interprofessional nature to it” (F2U2Y5Med2),

“We do get a bit of [interprofessional learning] with the nurses now… but we don’t get very much, we get an afternoon which is I don’t think long enough” (F3U2Y4Med).

Pharmacy students had similar experiences:

“It’s always just been like an afternoon with the medics… you’re not really developing any relationships with the other professions, it’s still very surface level and it’s still very much the medics and the pharmacists… it’s all still very separated like there’s not much integration” (F2U2Y4Pharm).

Finally, students reflected how future interprofessional learning might be embedded more effectively across their programmes to attain the learning they had experienced as part of the co-design process. Underpinning these suggestions were the principles of building relationships at a point when it might influence their practice:

“I think it needs to be from the start of university like you’re really building relationships like constantly every month’, "even just having tutorial groups… where like you’re kind of randomly blended in with all the courses” (F2U2Y4Pharm).

“Maybe tutorial groups of like 20 people… just so like relationships can build” (F2U1Y3Nurs1).

“Maybe like [working through] a scenario or something and then like you have to all work together to do that” (F2U2Y2Nurs).

Medical students who had experience of interprofessional learning early in their programme felt their experience might be more beneficial later in their programme:

“It’s so much more beneficial at this stage [4th year of medicine programme]” (F3U2Y4Med),

“Now we’re a little bit more advanced, we’re probably a little bit more in our roles… it’s probably a lot more beneficial to do it now, you’re going to get a lot more out of it now than early on” (F2U2Y5Med2),

“It doesn’t really work [early in course, because] neither students have been off on placement… whereas now people have got distinctive separate knowledge and distinctive separate experiences” (F1U2Y5Med2).

While each discipline had differing experiences of interprofessional learning, all students across both focus groups felt their exposure to interprofessional collaboration as part of the co-design process had laid the foundations for them to work more effectively with other disciplines to improve patient care.

#### Theme 3 – professional growth

Throughout all focus groups, students discussed areas of learning leading to professional growth as a result of their participation. For example, learning from each other:

“it’s been great… being able to learn off everybody and get their little bits and bobs of information” (F1U2Y1Nurs),

“[another participant] was using abbreviations and stuff and I was like okay right, that’s what that means… it’s handy to know going forward” (F1U2Y2Nurs).

One student also discussed an experience of delirium while working in a hospital between the first and second workshops which was benefited by their learning of delirium during the first workshop:

“I was [working with] a patient who was like, from my perspective, very obviously delirious… I had to bring it up to the doctors” (F2U1Y3Nurs2).

As well as learning, students also discussed areas of personal growth as a result of their participation. For example, increased confidence specifically with interacting with other disciplines:

“I think it’s given me a bit of… confidence” (F3U2Y4Med).

“Even like building confidence, like working with other… discipline”*’* (F2U2Y4Pharm).

In addition, students found being able to experience new things as part of the process beneficial:

“It’s been great to get the opportunity… people have got to do the podcast and being involved in filming… using an autocue… be involved in experiences that we’d never have had before or might get in the future” (F2U2Y4Pharm).

Overall, students highlighted many benefits of the co-design process which included the opportunity for their voice to be heard in the design of educational interventions for future students, opportunities to collaborate with students of different levels and disciplines, as well as areas of personal growth. It is evident that students gained an appreciation, especially, for interprofessional collaboration within their education and would like to see such collaboration more frequently incorporated into their programmes.

## Discussion

The present study utilised co-design methodology to develop an interdisciplinary health professions digital education resource. This resource is intended to address the lack of knowledge on the diagnosis and management of delirium which exists within health professionals [6, 8]. The co-design process provided an opportunity for healthcare profession students to collaborate and learn from each other as they co-created the resource. The interprofessional collaboration facilitated by the co-design process resulted in increased knowledge of others’ roles leading to reports of increased confidence, improved communication, and teamwork. Similarly, co-design has enhanced communication and collaboration between students in previous research [18–21]. Although the student experience of co-design was positive, inviting students into the co-design process where they may not be given specific direction may be overwhelming. Therefore, as this study has shown, the opportunity to build relationships prior to or as part of the co-design process is vital for the student experience.

The majority of feedback regarding the co-design format was positive. Students expressed surprise at the efficiency of the process and were pleased that the final outcomes reflected their student-voice. It is encouraging that students identified the need for executive decisions from the research team while still feeling the student input remained the major influence. This decision was in part due to conflicting schedules across institutions and between disciplines as, for example, some students were in practice while others were in class, a recognised barrier of interprofessional learning [15]. Additionally, many students in the group were coming to the end of their programme, with added work pressures, which reminded the research team that co-design is not co-working, and highlighted the balance between expertise, power, and authority between the research team and students which needs to be considered within co-design [32]. As discussed by the students in this study, there was a point in which students welcomed decisions made by the research team whilst still feeling their voices remained central to the process. This response from students demonstrates the value of this student/researcher partnership and efforts made to ensure students felt empowered to voice their opinions throughout the co-design process [19, 28].

The success of the co-design process in this study may have been facilitated by providing students with an intensive opportunity external to the curriculum but relatable back to their own area of practice. The opportunity for students to engage in an interprofessional learning activity external to their curriculum helped to address the discussed barriers to interprofessional learning [15]. In addition, the intensiveness of the experience, working together with other disciplines for a prolonged period of time may have facilitated both passive and active learning experiences, both of which have been shown to improve students’ attitudes towards interprofessional learning [33].

Given the importance of interprofessional collaboration within delirium care [13, 14], it is promising that students acknowledged the value of interprofessional collaboration following participation in the first co-design workshop. Further, this study highlights how the co-design process fosters interprofessional collaboration resulting in an increased understanding of each discipline’s role, improved communication and teamwork. Similarly, interprofessional learning aims to foster mutual respect and trust between disciplines [34], which was reflected as an outcome of the co-design process. The importance of acknowledging that each healthcare profession was working towards the same goal of patient care underpinned the value of interprofessional collaboration in this study. Further, students also expressed their belief that the continued involvement of students in the co-design of learning resources would bring positive benefits to both themselves and their professions.

Despite the acknowledged benefits of interprofessional learning [14, 15], which were further highlighted in this study, students also identified limited or ineffective interprofessional learning across their programmes. The focus of students in this study was the value of developing relationships early in their programme and then building on interprofessional learning opportunities as they progress through their programme. Previous research has suggested such education early in the undergraduate programme may help to prevent potentially negative stereotypes developing [35]. In addition, undergraduate healthcare students’ readiness for interprofessional education has been found to decrease as they progress through their programme and so early introduction is recommended to capitalise on the higher levels of readiness for interprofessional learning at the beginning of their studies [36].

Areas of professional growth were also evident. Students reported increased confidence in not only working with other disciplines but also due to the novel nature of co-creation of the resource though their involvement in podcasting and interprofessional simulation. Unlike most interprofessional learning opportunities, the recruitment of students to this study, included students at different stages in their programmes. Previous research has highlighted the benefits of peer learning, including increased confidence and team working skills, between students at different stages in their programmes [37] and between students of different disciplines [38]. The element of peer learning ensured students felt more confident about the recognition and management of delirium, resulting in one student having the confidence to assess and report a person with delirium in their practice placement between workshops. As the primary aim of this overall research project was to improve prevention, recognition, and management of delirium it is encouraging that those students who participated in the co-design phase have already shown increased ability to recognise the signs and symptoms of the condition.

### Implications for research, education and practice

This study suggests that students from different professions found the co-design process useful as an interprofessional learning activity. For interprofessional education to be meaningful, students need to see the application of their learning to practice, which the co-design of a learning resource achieved. Indeed, the students themselves suggested that they should be more involved in co-creating learning resources in similar ways. Whilst this research identified short term benefits in self-reported student learning, future research could consider the measurement of longer-term learning outcomes. This study reports anecdotal improvement in students’ practice as a result of the learning through the co-design process. Future research needs to consider how the co-design process might facilitate behaviour change in students that may lead to improved patient outcomes.

### Strengths and limitations of the co-design process

A strength of the co-design process used in the present study was the opportunity for students to work together in developing interprofessional case scenarios, filming simulated scenarios and recording podcasts resulting in the co-creation of the resource. However, it was also noted that some students felt the addition of more co-design tasks throughout the first workshop may have promoted efficiency. This is likely due to the number of relationship building activities incorporated into the first workshop which marked the first meeting of the student group and research team. However, the relationship building was an important aspect of the co-design process and facilitated the engagement reflected in later in the process.

Students identified a weakness of this study as a limited amount of allied health professions students. The present study included students from medicine, nursing, pharmacy, and occupational therapy from different year groups and found this to facilitate peer learning both between year groups and disciplines. Although other allied health groups were invited, the timing of the workshops did not facilitate wider involvement. Therefore, future research could explore the inclusion of additional allied health student groups in the codesign process (e.g. physiotherapy, dietetics).

## Conclusion

Across two co-design workshops healthcare profession students co-created the information and interactive features contained in the final digital resource. The engagement of different disciplines facilitated peer learning, increased confidence in working with other disciplines and a positive experience of interprofessional collaboration. This study demonstrates the effectiveness of co-design as a methodology for development of future interprofessional learning opportunities.

## Electronic supplementary material

Below is the link to the electronic supplementary material.

Supplementary Material 1

## Data Availability

The anonymised transcripts used and/or analysed during the current study are available from the corresponding author on reasonable request.
