# Supplementary material for: Co-designing an interprofessional digital education resource on delirium: a student-led approach
Source: BMC Med Educ. 2024 Oct 10;24:1122. doi: 10.1186/s12909-024-06023-8 (PMC11468056; doi:10.1186/s12909-024-06023-8)
Supplement: Supplementary file 1 — Supplementary Material 1 [file 12909_2024_6023_MOESM1_ESM.pdf]

Supplementary Material

Co-Design Workshop One Artefacts

S1: ‘What is Delirium?’ Stream of Consciousness Themes

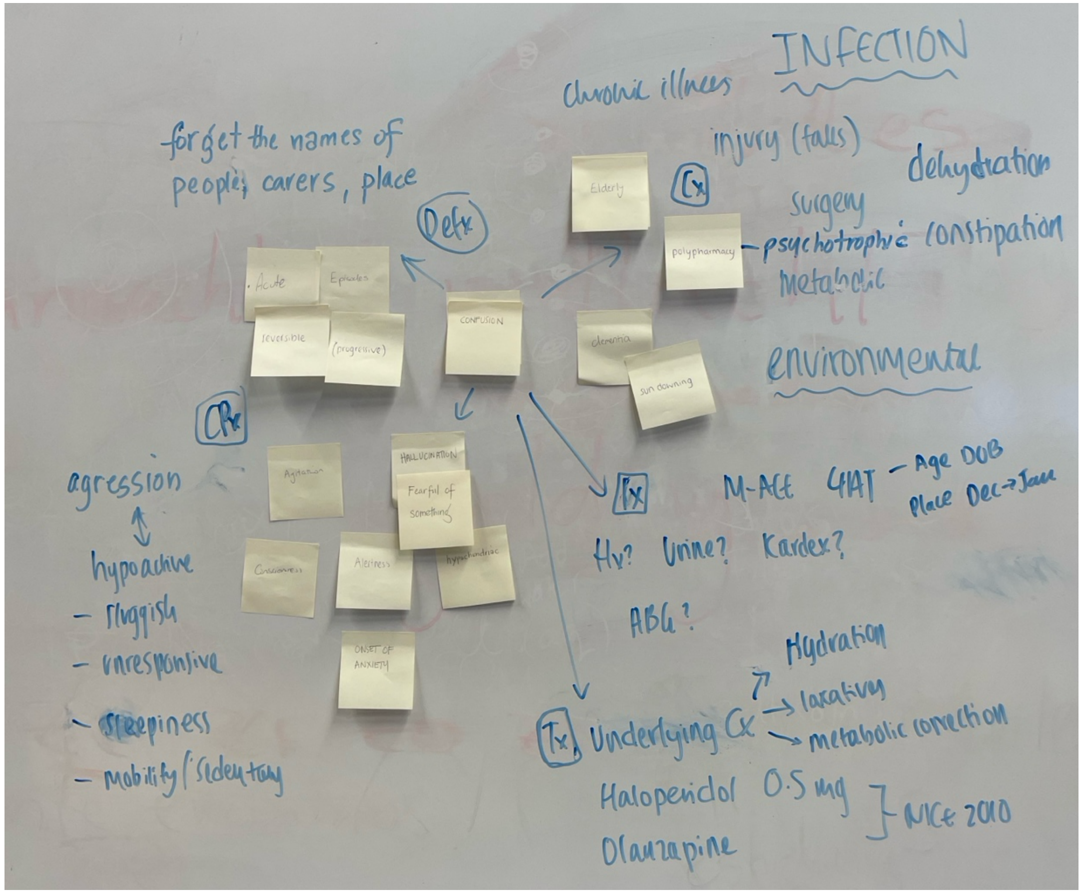

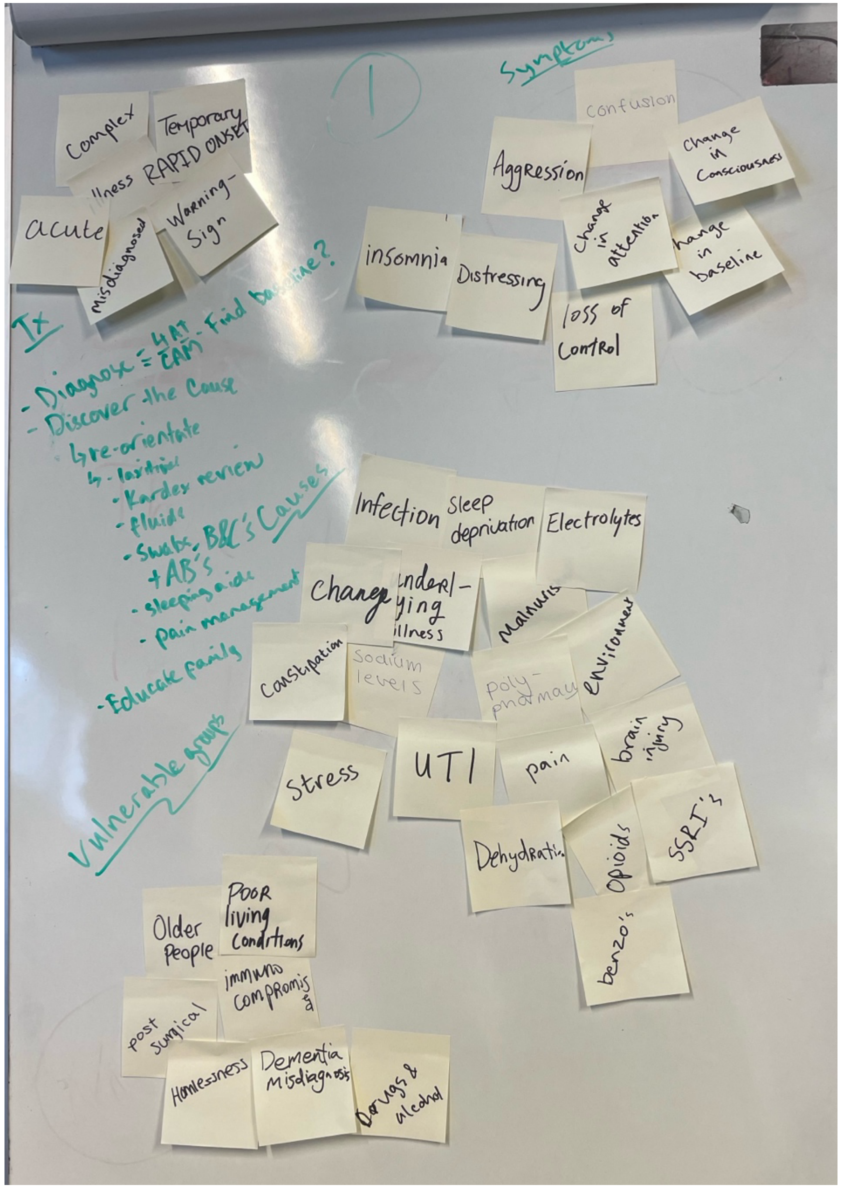

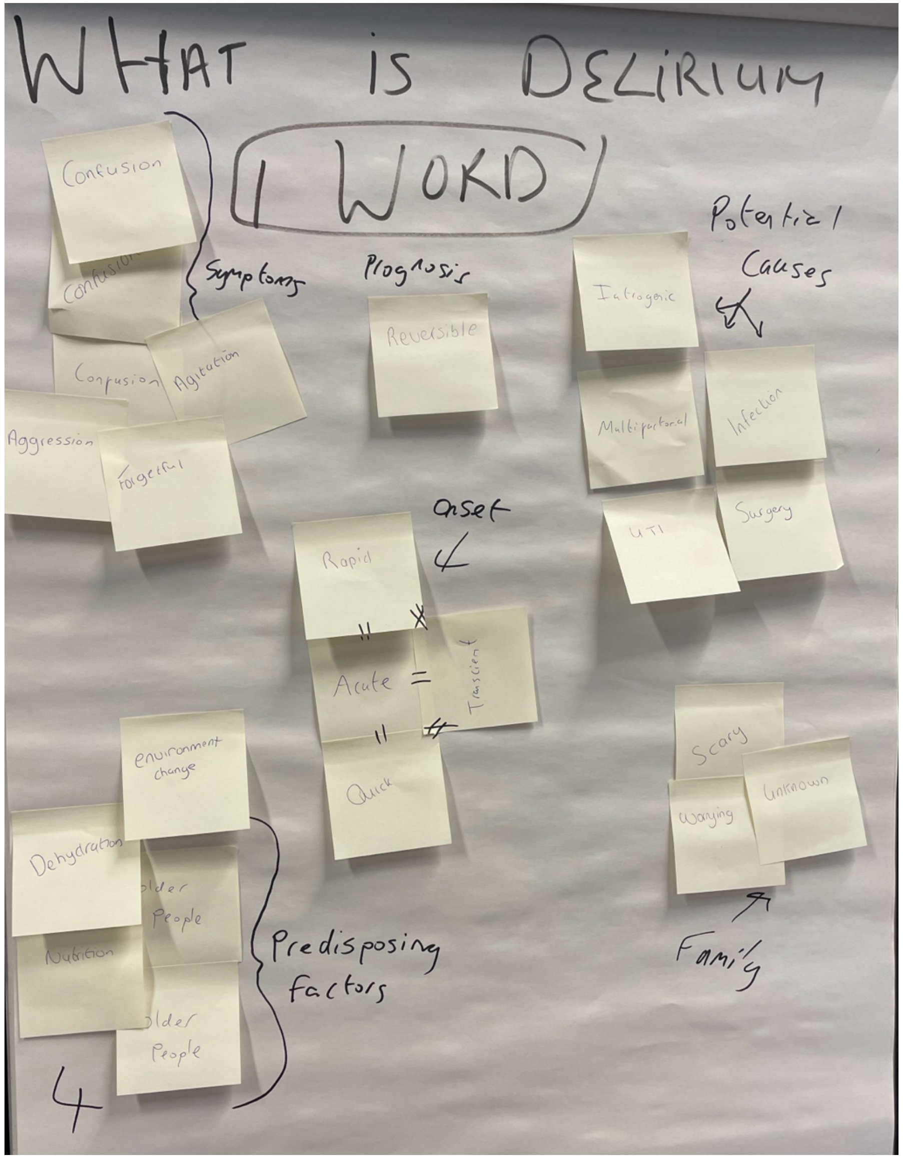
WHAT is DELIRIUM
1 WORD
Confusion
Confusion
Confusion
Agitation
Aggression
Forgetful
Symptoms
Prognosis
Reversible
Potential Causes
Iatrogenic
Multifactoral
Infection
UTI
Surgery
Onset
Rapid
Acute
Transient
Quick
environment change
Dehydration
Older People
Nutrition
Older People
Predisposing Factors
Scary
Worrying
Unknown
Family
4

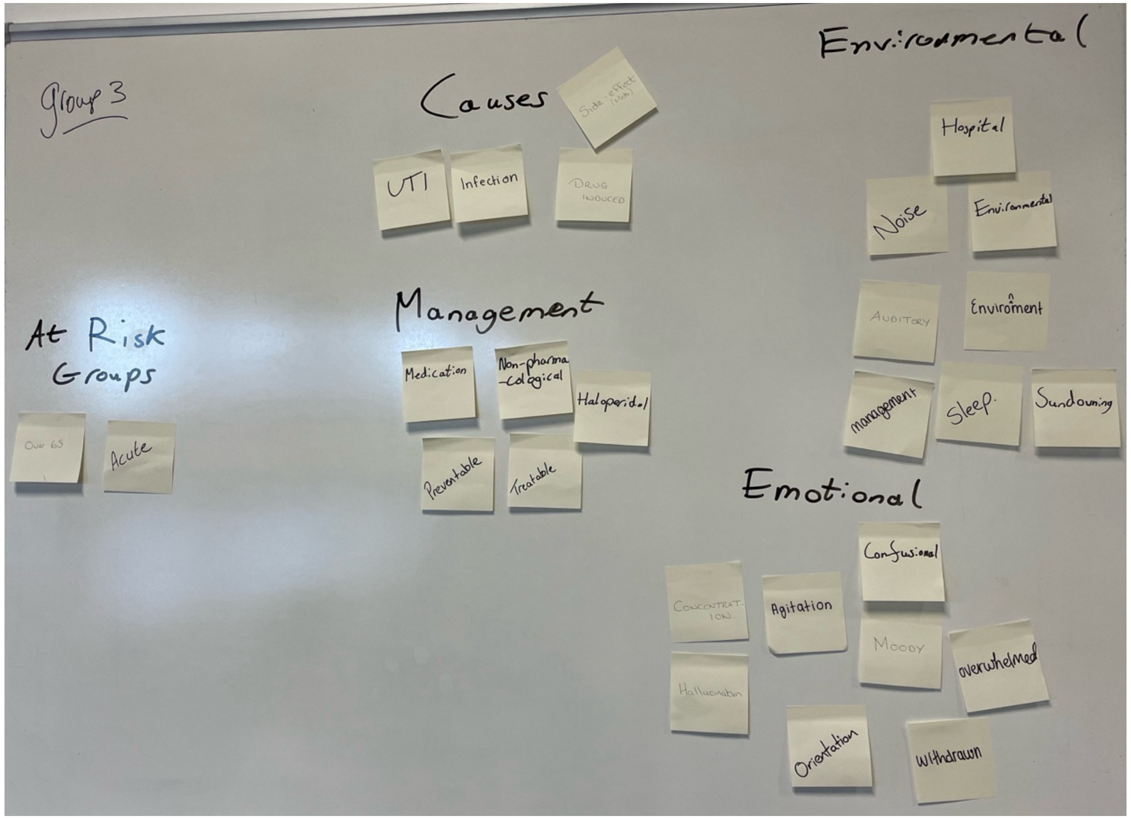
Group 3
Causes
Side effect
UTI
Infection
Drug induced
At Risk Groups
Acute
Management
Medication
Non-pharma-cological
Haloperidol
Preventable
Treatable
Environmental
Hospital
Noise
Environmental
Auditory
Enviroment
management
Sleep
Sundowning
Emotional
Confusional
Agitation
Moody
overwhelmed
Orientation
Withdrawn

S2: Scenario Development

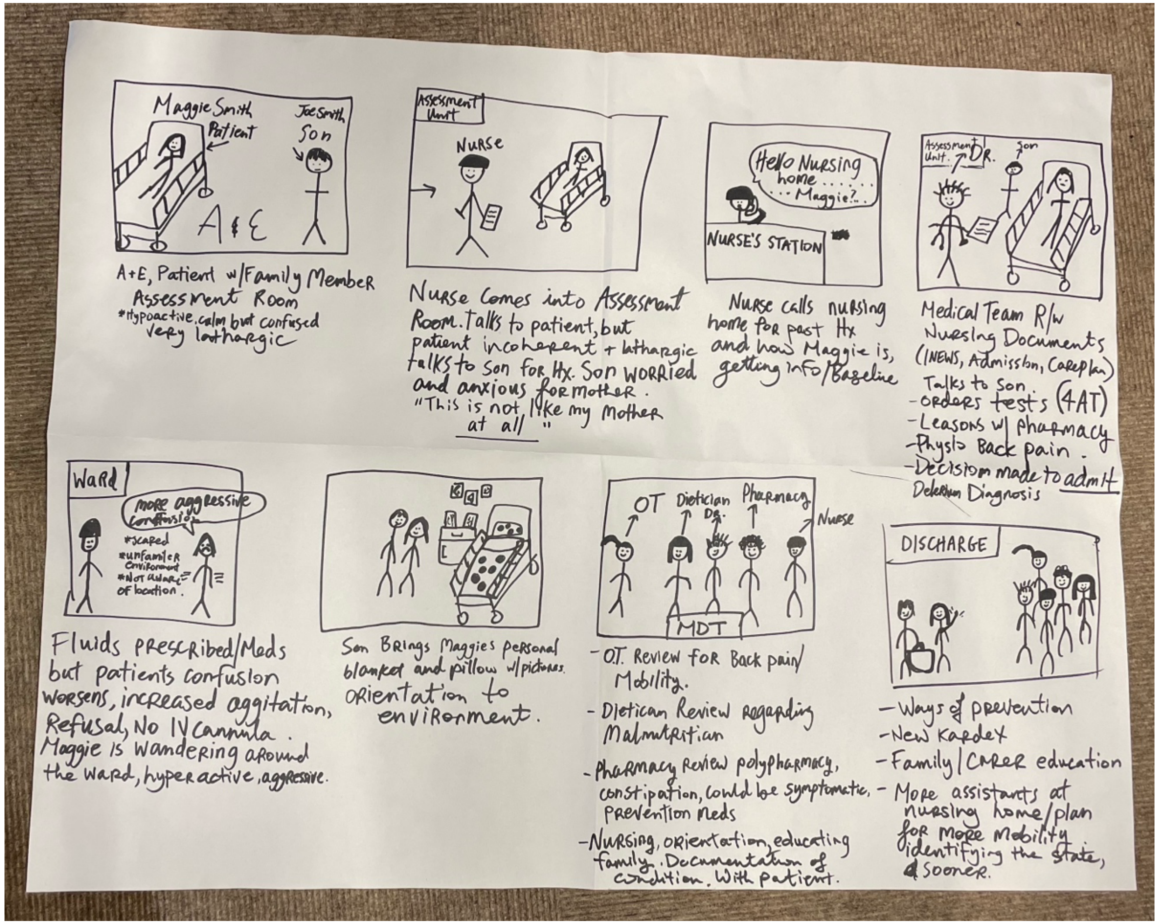

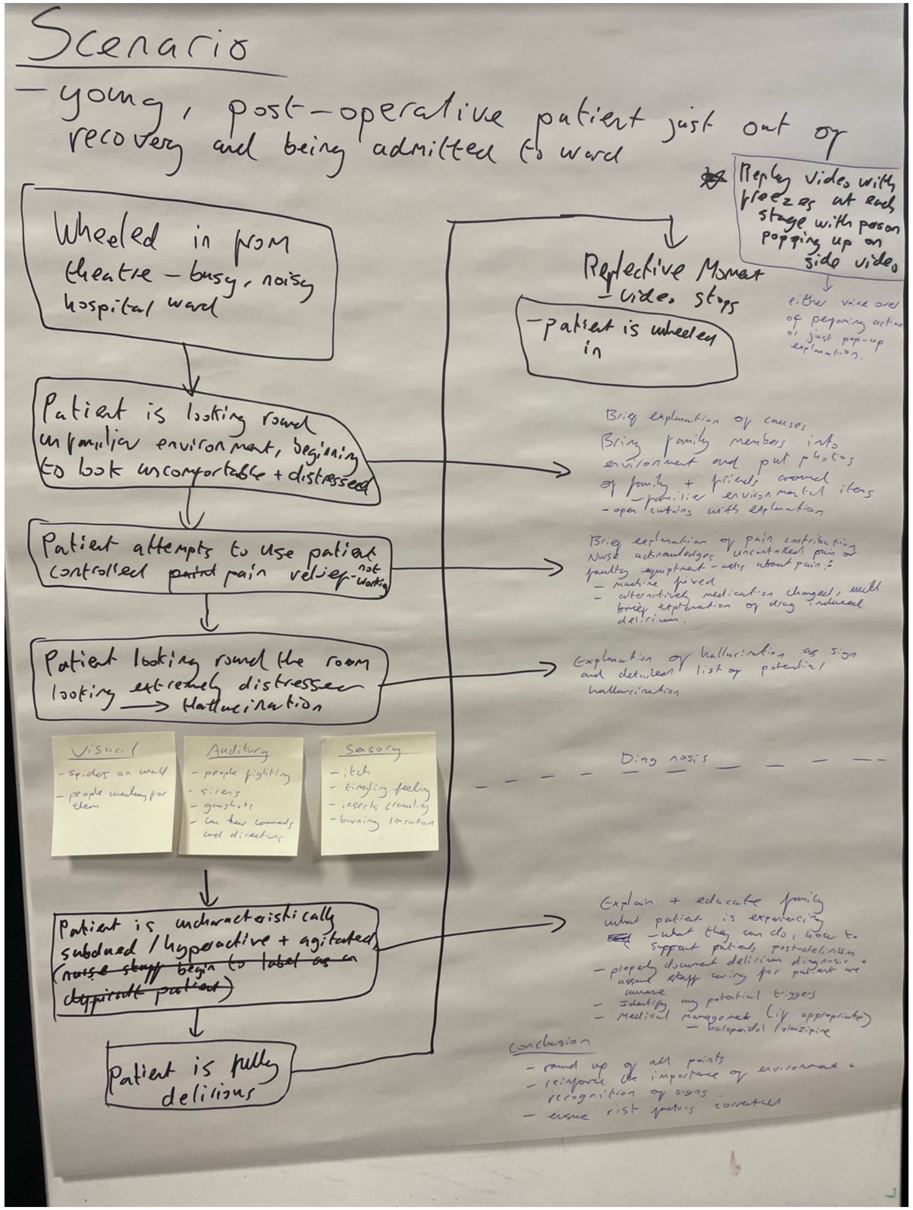

S3: Co-Design Workshop Two Artefacts

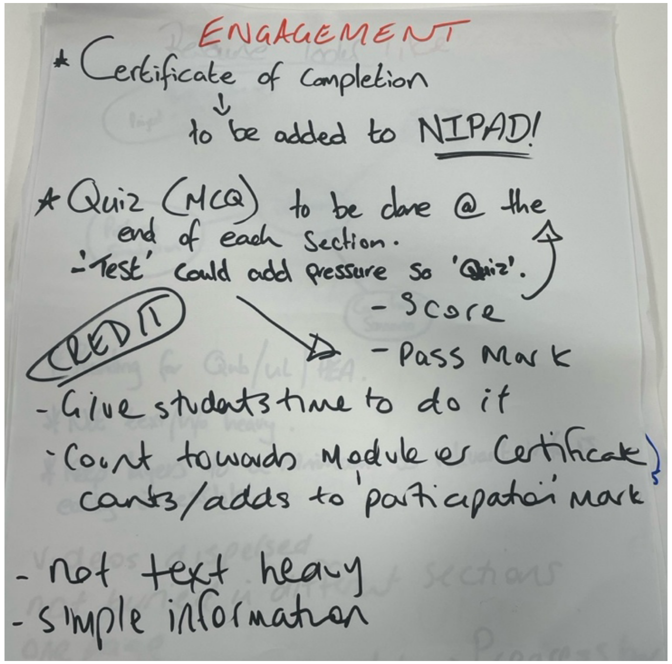

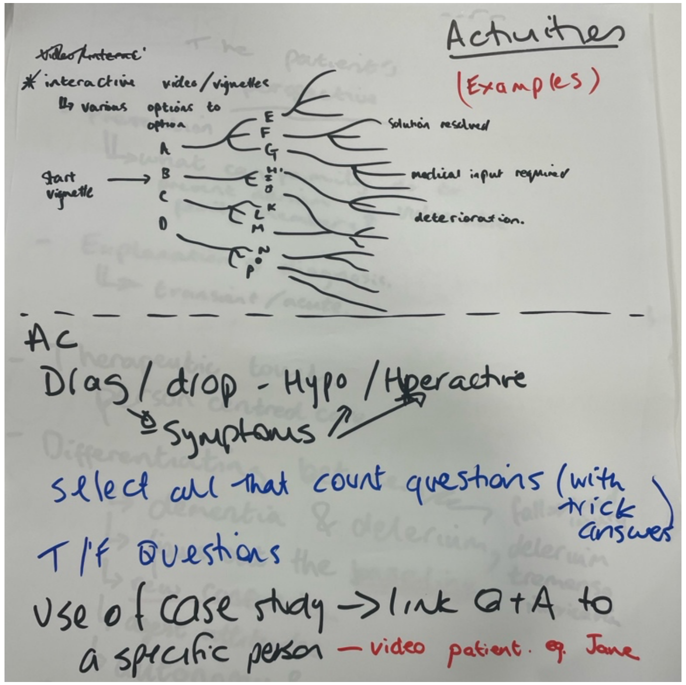

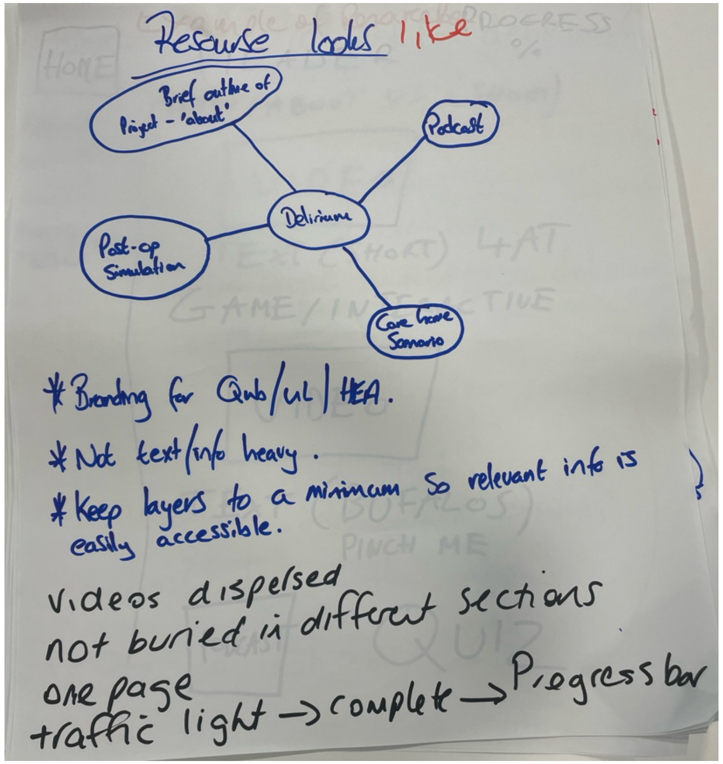

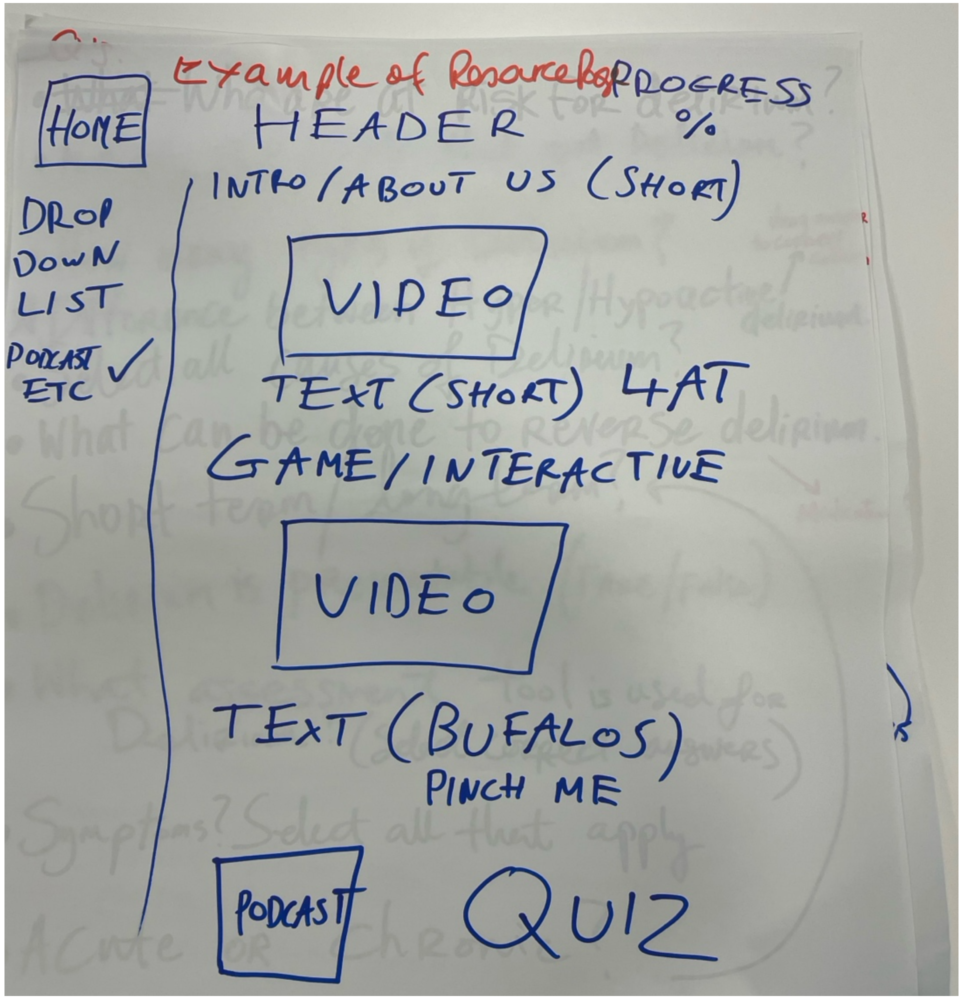

OTHER RESOURCES

- NICE
- BNF
- HSE Delirium document
- Project publications
- RTÉ 10 things to know about research
- PHA - delirium info for patients + relatives
- dementiapathways.ie
- Scottish Delirium Association
- Alberta Health Services Delirium + Dementia
